# Regulation of Locomotor activity in fed, fasted, and food-restricted mice lacking tissue-type plasminogen activator

**DOI:** 10.1186/s12899-018-0036-0

**Published:** 2018-01-25

**Authors:** Jessica A. Krizo, Linley E. Moreland, Ashutosh Rastogi, Xiang Mou, Rebecca A. Prosser, Eric M. Mintz

**Affiliations:** 1 Department of Biological Sciences, Kent State University, Kent, OH 44242 USA; 2 Department of Molecular and Cellular Biology, Baylor College of Medicine, Houston, TX 77030 USA; 3 Department of Biochemistry and Cellular and Molecular Biology, University of Tennessee, Knoxville, TN 37996 USA

**Keywords:** Circadian, Food anticipatory activity, Wheel-running

## Abstract

**Background:**

Circadian rhythms of physiology and behavior are driven by a circadian clock located in the suprachiasmatic nucleus of the hypothalamus. This clock is synchronized to environmental day/night cycles by photic input, which is dependent on the presence of mature brain-derived neurotrophic factor (BDNF) in the SCN. Mature BDNF is produced by the enzyme plasmin, which is converted from plasminogen by the enzyme tissue-type plasminogen activator (tPA). In this study, we evaluate circadian function in mice lacking functional tPA.

**Results:**

tPA^−/−^ mice have normal circadian periods, but show decreased nocturnal wheel-running activity. This difference is eliminated or reversed on the second day of a 48-h fast. Similarly, when placed on daily cycles of restricted food availability the genotypic difference in total wheel-running activity disappears, and tPA^−/−^ mice show equivalent amounts of food anticipatory activity to wild type mice.

**Conclusions:**

These data suggest that tPA regulates nocturnal wheel-running activity, and that tPA differentially affects SCN-driven nocturnal activity rhythms and activity driven by fasting or temporal food restriction.

**Electronic supplementary material:**

The online version of this article (10.1186/s12899-018-0036-0) contains supplementary material, which is available to authorized users.

## Background

Circadian rhythms of physiology and behavior are driven by a circadian clock located in the suprachiasmatic nucleus of the hypothalamus (SCN) [1, 2]. The SCN is directly innervated by retinal ganglion cells, which provide the entrainment signals that synchronize SCN rhythms with the environmental light-dark (LD) cycle [3–5]. The signal transduction pathway that conveys photic information to the SCN is dependent on the activation of the trkB receptor by brain-derived neurotrophic factor (BDNF) [6]. The production of the mature form of BDNF in the brain is at least partly dependent on the extracellular activity of tissue-type plasminogen activator (tPA), which converts plasminogen to plasmin, which in turn catalyzes the conversion of proBDNF to mBDNF [7]. Both BDNF and trkB are found in the SCN [6, 8, 9].

BDNF signaling deficits lead to a decrease in light induced phase shifts [10], and trkB antagonists in the SCN block light induced phase shifts of the circadian clock in vivo [11] and glutamate-induced phase shifts in vitro [9]. Further, tPA inhibition in vitro decreases glutamate-induced phase shifts [9]. These findings suggest that tPA activity is important for regulating glutamate induced phase shifts. Surprisingly, mice that lack tPA have normal free-running periods and normal phase-shifting responses to light pulses, though they do show slower entrainment to a large shift of the light-dark cycle [12]. This suggests that any deficiency in entrainment in these mice is modest. However, in the course of screening tPA knockout mice (tPA^−/−^) for their circadian phenotype, we noted that overall wheel-running activity appeared to be reduced compared to wild type mice. Mice that lack BDNF in adulthood are hyperactive [13], suggesting the possibility that depressed activity in tPA^−/−^ mice occurs via a BDNF-independent mechanism.

BDNF also has been implicated in regulating the brain’s adaptations to energetic challenges [14]. When food availability is restricted to a narrow window of time per day, rodents exhibit a behavior known as food anticipatory activity [15], which occurs for a 2-3 h period prior to food availability. This activity appears to be driven by a food-entrainable circadian oscillator, and persists in the absence of a functional SCN [16, 17] or critical components of the molecular circadian clock mechanism [18, 19]. A number of neuroendocrine regulatory factors contribute to the appearance of food anticipatory activity (for a review, see [20]), however, the underlying mechanisms are still poorly understood. Because the loss of tPA reduces neuronal plasticity, and due to its known, but limited effects on SCN entrainment pathways, we hypothesized that mice lacking tPA would have difficulty adapting to timed restricted feeding regimes.

## Methods

### Animals

Animals used in this study were age-matched across each experimental group in each study. Two to four-month old male C57BL/6 J wildtype mice (tPA^+/+^) and tPA knockout mice (tPA^−/−^) (bred from stock purchased from Jackson Laboratory (Bar Harbor, ME), backcrossed to C57BL/6 J) were used in all experiments. Variation in age is based on the length of study and animal availability, however, in all studies genotypes were age matched. No animals were used in more than one experiment. Animals were individually housed in Plexiglas cages equipped with a running wheel. Animals were housed at a temperature of 20 °C and had access to water ad libitum. Food was also available ad libitum except as indicated below. All animal use protocols in this study were approved by the Kent State Institutional Animal Care and Use Committee and were performed in accordance with the recommendations in the Guide for the Care and Use of Laboratory Animals of the National Institutes of Health.

### Assessment of mature BDNF in the SCN

To assess in vivo protein expression, SCN tissue was dissected from mouse brains at zeitgeber time (ZT) 4 (4 h after lights on) and ZT 12 and immediately frozen for later Western blot assay as described before [9]. Harvested tissue was homogenized in ice-chilled HEPES-based extraction buffer containing a protease inhibitor cocktail (1 mM phenylmethylsulfonyl fluoride, 10 mg/mL aprotinin, 15 mg/mL leupeptin, 10 mg/mL pepstatin). For BDNF immunoblots, tissue was prepared in Tris-based denaturing extraction buffer [4 M urea, 0.02 M dithiothreitol, 0.05 M Tris, pH 7.4, 2% sodium dodecyl sulfate (SDS)]. The tissue extract sample was separated into aliquots and stored at − 80 °C. Protein content of the extract was determined by the bicinchoninic acid method (BCA; Pierce). Tissue samples were mixed with loading buffer (pH 6.8 Tris, SDS, bromophenol blue, glycerol), and subjected to SDS–polyacrylamide gel electrophoresis. Proteins were electrotransferred onto nitrocellulose membranes, which were then incubated with blocking buffer [10% solution of non-fat dry milk in phosphate-buffered saline with Tween-20 (PBST, 8 mM Na_2_HPO_4_, 150 mM NaCl, 2 mM KH_2_PO_4_, 3 mM KCl, 0.05% Tween-20, pH 7.4)]. The membranes were probed with primary antibodies diluted in a 2.0% solution of non-fat dry milk in PBST, followed by goat anti-rabbit horseradish peroxidase-conjugated secondary antibody at appropriate dilutions in the same buffer. Signals were revealed by enzyme-catalyzed chemiluminescence (Pierce, IL, USA). The amount of protein loaded in each lane was assessed by probing for α-tubulin, so all protein expression was calculated as levels relative to α-tubulin. Control experiments included running parallel lanes loaded with the corresponding native proteins (positive controls), and probing membranes with primary antibodies pre-incubated with the native protein to test for cross-reactivity and to establish the specificity of the antibody samples. Rabbit anti-α-tubulin antibodies were obtained from Santa Cruz Biochemicals (Santa Cruz, CA, USA). Rabbit anti-proBDNF antibody was obtained from Millipore (MA, USA) and rabbit anti-BDNF antibody was from Alomone Labs (Jerusalem, Israel). We differentiated between pro- and mBDNF proteins by using respectively specific antibodies as well as by their distinct sizes. proBDNF was identified as ~37kD bands, while mBDNF ~15kD bands.

### Restricted feeding

Following a two-week baseline activity recording period, mice were deprived of food for 48 h. Mice were then given four days of free food access before food removal at lights off (ZT 12). Subsequently, food was presented to mice at ZT 6 and removed at ZT 10. This was continued for eight to ten days at which point experimental protocol varied as detailed below. Body weight was measured during baseline activity, following fast, following free feeding period and after restricted feeding.

### Activity measurement

All cages were equipped with either traditional stainless steel 6 in. diameter running wheels or 6.10 in. running wheel discs. Traditional running wheel data was collected as revolutions per minute with ClockLab (Actimetrics, Wilmette, IL) and running disc data was collected with Med Associates (St. Albans, VT); data was qualified and quantified using ClockLab. Experiments in regular LD cycles were performed using the running wheels and the skeleton photoperiod data in the supplementary data file used the running discs. Due to differences in data collection between wheels, comparisons between studies using different wheels were not made. The use of two different wheel systems was necessary in order to complete the studies in a timely manner. Activity profiles were calculated as an average activity per animal and per genotype as follows: baseline, averaged 4 day baseline and restricted feeding averaged over days three through eight. Activity profiles were created using total revolutions per hour as a percentage of total 24 h baseline activity. Food anticipatory activity (FAA) was defined as activity measured during the 3 h (ZT 3-6) prior to food presentation. This 3 h period was chosen based on a review of the FAA literature and to provide for a consistent measurement interval.

### Assessment of baseline activity profiles in a 12:12 LD cycle

Mice were housed in a 12:12 LD cycle and activity profiles were assessed, both in absolute terms and as a percentage of the 24-h mean for each animal.

### Food anticipatory activity in tPA^−/−^ mice under light-dark conditions

Male tPA^+/+^ and tPA^−/−^ mice were maintained in a standard 12:12 light-dark cycle and underwent the food restriction protocol described above.

### Assessment of circadian phase during food restriction

Male tPA^+/+^ and tPA^−/−^ mice were placed in a skeleton photoperiod after being entrained to a 12:12 light-cycle. Mice were divided into four groups, tPA^+/+^ RF and tPA^−/−^ RF were food restricted and tPA^+/+^ and tPA^−/−^ ad/libitum feeding groups (AL) had continuous access to food. After ten days of restricted feeding, mice were released into constant dark conditions with continuous access to food. Mice were allowed to free run for two weeks before phase was measured.

### Food intake changes during restricted feeding

Male age matched tPA^+/+^ and tPA^−/−^ mice were individually housed in small Plexiglas cages with metal grated cage liners and a PVC pipe for comfort. Weight and food intake was measured daily to generate a baseline. Mice then were food restricted and weight and food intake was measured daily. Body composition analysis was completed with the use of an EchoMRI (Echo Medical Systems, Houston, TX) for baseline, after fast, and before and after restricted feeding. EchoMRI measured fat mass and lean mass, with lean mass being calculated as total body mass minus fat mass.

### Statistical analysis

Analyses were performed using NCSS 10 software (Kaysville, UT). Comparisons between groups were performed using one-way and two-way ANOVA with repeated measures where appropriate. Planned comparisons between genotypes throughout the 24-h cycle were assessed using Fisher’s LSD test if the ANOVA showed a statistically significant interaction between genotype and clock time. Fisher’s LSD is used because the time-series data being analyzed have a strong serial correlation and this results in most other tests being overly conservative. Significance was ascribed if *p* < 0.05.

## Results

### Assessment of mBDNF/proBDNF ratio in the SCN

**Fig. 1:**
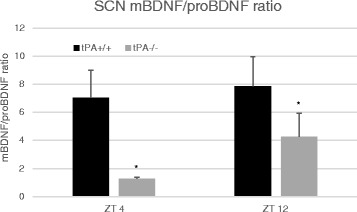
mBDNF to proBDNF Ratio. Ratio of mBDNF to proBDNF at ZT4 and ZT12, in tPA^−/−^ and tPA^+/+^ mice. There is a significant (*p* < 0.05) main effect of genotype, with tPA^−/−^ mice having a reduced ratio compared to tPA^+/+^ mice. All groups have a sample size of 3. Error bars represent standard error of the mean

First, we established whether there was a reduction in mBDNF levels in the SCN of tPA^−/−^ mice. For these experiments, SCN tissue was isolated from tPA^+/+^ and tPA^−/−^ mice at ZT 4 and ZT 12 and immediately transferred into extraction buffer for protein analysis. SCN content of pro- and mBDNF from tPA^−/−^ and tPA^+/+^ mice was quantified and normalized to α-tubulin. Then an mBDNF to proBDNF ratio was computed as the index for relative mBDNF quantity. There was a significant main effect of genotype (F_1,8_ = 7.88, *p* = 0.023) (Fig. 1), but not ZT (F_1,8_ = 1.29, *p* = 0.29) or an interaction (F_1,8_ = 0.42, *p* = 0.53), indicating reduced conversion of proBDNF to mBDNF in tPA^−/−^ mice.

### Reeintrainment to an advance of the LD cycle

**Fig. 2:**
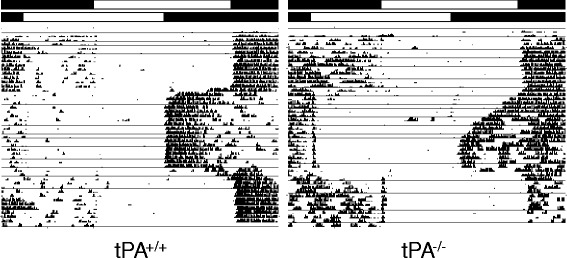
Simulated Jet Lag. Representative actograms showing a 6 h advance and a 6 h delay of the light-dark cycle in both genotypes

We previously reported that tPA^−/−^ mice took longer to adjust to a 12-h shift of the LD cycle than did tPA^+/+^ mice [12]. However, these shifts represent only the phase delaying effects of light. To measure the impact of the loss of tPA on phase advances, we measured the time to adjust to a 6-h advance of the LD cycle (Fig. 2). tPA^−/−^ mice took significantly longer (8.1 ± 0.7 days) than tPA^+/+^ (5.9 ± 0.5 days) to reentrain to the shifted LD cycle (t_17_ = 2.57, *p* = 0.02). We also exposed the animals to a 6-h phase delay, but due to suppression of activity by light (masking) an accurate assessment of reentrainment time could not be performed.

### Locomotor activity during timed restricted feeding

**Fig. 3:**
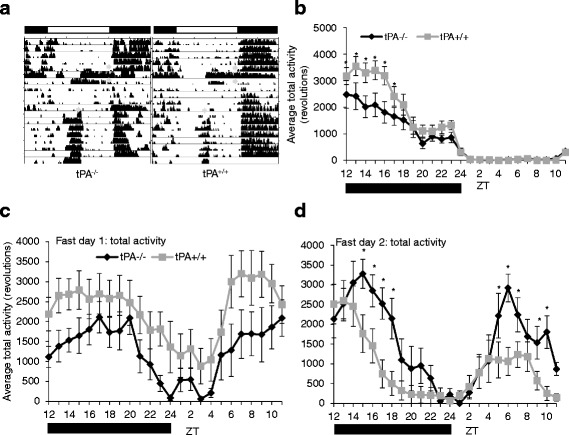
Activity During LD – Baseline and Fasting. Representative actograms (**a**) of food restriction protocol in tPA^−/−^ and tPA^+/+^ mice in LD. Arrows indicate onset and offset of fast and beginning of restricted feeding. Fast includes food removal at ZT 12 and return 48 h later at ZT 12. Average 24 h activity profile (**b**), bar indicates dark phase; locomotor activity is higher in tPA^+/+^ (*n* = 13) than tPA^−/−^ (*n* = 14) at ZT 12-17 (*p* < 0.05). During fast day 1 total locomotor activity (**c**) is higher in tPA^+/+^ (*n* = 8) than tPA^−/−^ (*n* = 7) (*p* < 0.05) with no effect of time of day. During fast day 2 (**d**) total locomotor activity is significantly higher in tPA^−/−^ (*n* = 8) than tPA^+/+^ (*n* = 8) during both the night (ZT15-18) and the day (ZT 5-7, 9-10) (*p* < 0.001). Error bars represent standard error of the mean

**Fig. 4:**
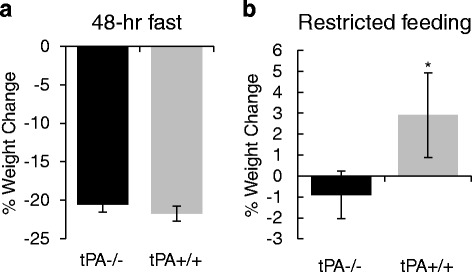
Body Weight Comparison. Weight changes between genotypes are consistent following a 48-h fast (**a**); following restricted feeding (**b**) tPA^+/+^ increased in weight more than tPA^−/−^ (*p* < 0.05). Sample sizes are 15-16 per group. Error bars represent standard error of the mean

**Fig. 5:**
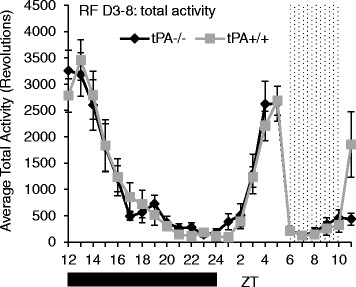
Activity During LD Restricted Feeding. Activity profile averages from days 3 through 8 of restricted feeding. Bars indicate the dark period and dots indicate food availability from ZT6-10. Total locomotor activity is not different between genotypes during restricted feeding. Samples sizes are 13 per group. Error bars represent standard error of the mean

During baseline measurements both tPA^+/+^ and tPA^−/−^ mice exhibit typical patterns of nocturnal locomotor activity. Nocturnal activity was divided into two discrete bouts of locomotor activity, a high level of activity in early to mid-night ending in a drop of locomotor activity followed by a brief increase in activity ending gradually at ZT 24. However, the level of activity was reduced in tPA^−/−^ mice during the first part of the dark phase in LD (Fig. 3a and b) from ZT12-17 (F_23,575_ = 2.63, *p* < 0.001). Food availability had an effect on both the pattern and level of locomotor activity in tPA^+/+^ and tPA^−/−^ mice. Food deprivation led to increased diurnal activity across genotypes on both days. When food was removed at ZT 12 locomotor activity was suppressed compared to baseline activity during the first portion of the dark phase. tPA^−/−^ mice had decreased activity compared to tPA^+/+^ mice on LD fast day one (F_1,22_ = 4.57, *p* = 0.044)(Fig. 3c). During fast day two locomotor activity increased significantly over tPA^+/+^ (Fig. 3d) during both night (ZT 15-18) and day (ZT 5-7, 9, 10) (F_23,529_ = 2.23, *p* < 0.001). There was no difference in weight loss between genotypes (Fig. 4a) (tPA^−/−^: − 20.6% ± .008 and tPA^+/+^: − 21.7% ± .009, t_29_ = 0.937, *p* = 0.399). During restricted feeding the baseline differences in raw locomotor activity between genotypes disappeared (Fig. 5) and there was no difference in nocturnal or food anticipatory activity levels (F_23,547_ = 1.18, *p* = 0.253). Following restricted feeding tPA^−/−^ mice gained less weight than tPA^+/+^ mice, but this difference was not statistically significant (Fig. 4b)(tPA^−/−^: −.9% ± .01 and tPA^+/+^: 2.91% ± .02, t_28_ = − 1.65, *p* = 0.109).

This experiment was repeated, except that the light cycle utilized was a skeleton photoperiod, which is 15 min of light only at the beginning and end of a 12 h “day”. This design examines whether activity during the day is being suppressed, or “masked”, by the presence of light. The results from this experiment did not substantially differ from those obtained in standard light/dark conditions (Additional file 1).

### Assessment of circadian phase during food restriction

**Fig. 6:**
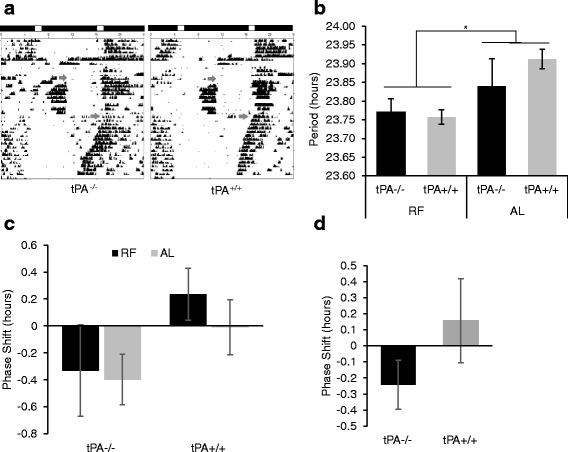
Free-running Activity After Restricted Feeding. Representative actograms of fasting and food restriction protocol in tPA^−/−^ and tPA^+/+^ (**a**) in skeleton photoperiod, followed by release into constant dark (indicated by arrow). There is no genotypic difference in period (**b**), however it there is a significant shortening of free-running period after RF as compared AL (*p* < 0.05, sample sizes of RF-tPA^−/−^: 9; RF- tPA^+/+^: 11; AL-tPA^−/−^: 3; RF- tPA^+/+^: 4*)*. Upon transfer to DD and ad/lib feeding, the unmasked phase of the underlying nocturnal activity rhythm did not differ between genotypes (sample sizes of tPA^−/−^: 9; tPA^+/+^: 7) in either LD_sk_ (**c**) or LD (**d**). Error bars represent standard error of the mean

The effect of timed restricted feeding on the SCN can be masked by light. When released to constant darkness following restricted or ad libitum feeding there was no genotypic effect on free-running period (F_1,23_ = 0.60, *p* = 0.447) (Fig. 6a and b). However, RF treatment had an aftereffect on free-running period, shortening the free-running period of RF groups (tPA^−/−^ RF: 23.77 ± 0.036, tPA^+/+^ RF: 23.75 ± 0.019) compared to AL groups (tPA^−/−^ AL: 23.84 ± 0.073, tPA^+/+^ AL: 23.91 ± 0.026) (F_1,23_ = 8.49, *p* = 0.008). Activity data were also analyzed to determine if the underlying nocturnal activity rhythm was advanced in food restricted mice, which would not be observable in LD due to the masking effect of light on activity but which could subsequently be predicted by the onsets of activity upon release into DD. There was no evidence of an underlying shift in the phase angle of entrainment toward food presentation in LD_sk_ across genotypes (F_1,23_ = 2.24, *p* = 0.148) or treatment (F_1,23_ = 0.024, *p* = 0.631) (tPA^−/−^ RF: −.33 ± 0.339, tPA^+/+^ RF: .24 ± 0.194, tPA^−/−^ AL: −.40 ± 0.188, tPA^+/+^ AL: −.01 ± 0.203) (Fig. 6c). Additionally, no difference in phase angle of entrainment was seen between genotypes following release from RF in LD to constant conditions (tPA^−/−^ RF = − 0.24 ± 0.154, tPA^+/+^ RF = 0.15 ± 0.262)(t_14_ = 1.383, *p* = 0.1882) (Fig. 6d).

### Food intake analysis

**Fig. 7:**
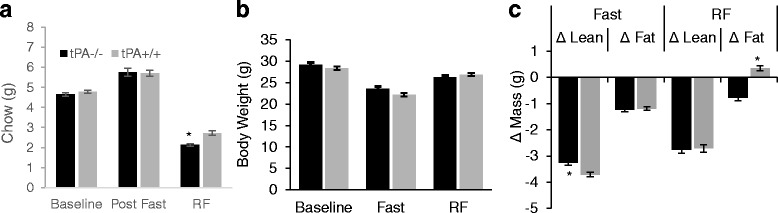
Food Intake and Body Composition. There is no difference in food intake (**a**) at baseline or following food deprivation. During RF tPA^−/−^ consume less chow than tPA^+/+^ *(p < 0.05*). There are no differences in body weight (**b**) at any part of the study. Analysis of body composition over the course of the restricted feeding protocol (**c**) shows no difference in changes in lean or fat mass during the 48-h fast. During restricted feeding lean mass change is consistent between genotypes but fat mass change is different between genotypes (*p* < 0.05). Sample sizes for all groups are 10. Error bars represent standard error of the mean

Since differences in FAA might reflect differences in the motivation for feeding, we compared food intake in tPA^−/−^ and tPA^+/+^ mice. There was no difference in food intake (tPA^−/−^: 4.65 g ± .09 g tPA^+/+^: 4.79 g ± .07 g) during standard LD conditions with food available ad libitum (t_18_ = 0.045, *p* = 0.964). During ad libitum feeding following food deprivation there was no difference in food intake (tPA^−/−^: 5.76 g ± .19 g tPA^+/+^: 5.70 g ± .15 g) (t_18_ = 0.21, *p* = 0.83). During restricted feeding food intake in tPA^−/−^ mice was reduced compared to tPA^+/+^ (tPA^−/−^: 2.14 g ± .06 g, tPA^+/+^ 2.73 g ± .11 g) (t_18_ = 4.656, *p* < 0.001) (Fig. 7a). There are no significant genotypic differences in weight at baseline (tPA^−/−^ = 29.21 g ± .58 g, tPA^+/+^ = 28.43 g ± .44 g) (t_18_ = 1.073, *p* = 0.297 or following RF (tPA^−/−^ = 26.27 g ± .41 g, tPA^+/+^ = 26.91 g ± .47 g) (t_18_ = 1.031, *p* = 0.316) (Fig. 7b). While tPA^−/−^ mice weighed slightly more than tPA^+/+^ after 48 h of food deprivation, the difference was not statistically significant (tPA^−/−^ = 23.62 ± .51, tPA^+/+^ = 22.22 ± .44) (t_18_ = 2.081, *p* = 0.0519). Changes in weight following food deprivation were due to changes in lean mass regardless of genotype. tPA^−/−^ mouse lean mass change was less than tPA^+/+^ (tPA^−/−^ = − 3.265 g ± .23 g, tPA^+/+^ = − 3.7076 g ± .27 g) (t_18_ = 3.919, *p* = 0.001). There was no difference in the loss of fat mass between genotypes (tPA^−/−^ = 1.243 g ± .11 g, tPA^+/+^ = − 1.1872 g ± .20 g) (t_18_ = − 0.453, *p* = 0.657). Following RF there was no genotypic difference in lean mass change (tPA^−/−^ = − 2.753 g ± .10 g, tPA^+/+^ = − 2.714 g ± .45 g)(t_18_ = 0.223, *p* = 0.823) but fat mass change differed between genotypes (tPA^−/−^ = −.78 g ± .26 g, tPA^+/+^ = .3533 g ± .10 g) (t_18_ = 4.097, *p* < 0.001) (Fig. 7c).

## Discussion

This research was initiated with the goal of examining the role of tPA in regulating circadian rhythms of activity, particularly with regard to circadian clock-driven responses to restricted feeding. The results, however, suggest a more subtle role for tPA in modulating the circadian rhythm of locomotor activity output. The loss of tPA should have a significant impact on the ability of animals to entrain to light cycles, through reduced BDNF levels [6, 9]. However, recent data suggests that urokinase-type plasminogen activity may substitute for tPA in the tPA^−/−^ mice [12]. Our initial finding was that tPA^−/−^ mice had reduced nocturnal wheel-running under a standard 12:12 LD cycle. It is likely that this results from the deficiency in mature BDNF in these mice. The positive link between BDNF and locomotor activity has been examined largely in the context of animal models of depression [21, 22], but not explicitly for the motivated behavior of voluntary wheel-running. However, we might have expected tPA^−/−^ mice to show deficits in circadian entrainment, given the reduction in mBDNF and the importance of mBDNF to the circadian clock’s photic signaling system [6, 10]. We interpret this to mean that the residual mBDNF remaining is sufficient to allow for normal entrainment of the circadian clock by light. This residual BDNF is produced by the activation of plasmin by enzymes other than tPA, such as urokinase-type plasminogen activator [12] or other enzymes that perform this function such as kallikreins [23].

Interestingly, reduced wheel-running in the tPA^−/−^ mice was reversed on day two of a 48-h fast. During fasting, both tPA^+/+^ and tPA^−/−^ mice show a second daily peak of activity during the light phase of the light-dark cycle. On day one of the fast, activity is elevated during the light phase in both genotypes, but is still reduced in tPA^−/−^ mice. On day two, however, nocturnal wheel-running in tPA^+/+^ mice is reduced while it is increased in tPA^−/−^ mice, suggesting an increased activity in response to the energetic deficit. The timing of the behavior also seems to be somewhat altered on day two, with the peak in dark-phase locomotor activity delayed slightly in tPA^−/−^ mice. Despite the difference in locomotor activity, however, there was no difference in total weight loss during the fast. The phenomenon of fast-induced increases in activity in rodents is well documented [24], however, our data suggests a role for tPA in the neural processes that regulate this behavior, in a manner distinct from circadian clock-mediated locomotor activity. Furthermore, the increase in wheel-running in tPA^−/−^ mice on day 2 of the fast provides evidence that the decrease in wheel-running under baseline conditions is not due to any kind of physical deficiency, but is more likely related to processes relating to the motivation for wheel-running.

Because of the role tPA plays in regulating plasticity in the brain [25], we had anticipated that tPA^−/−^ mice might have some difficulty adapting to a timed restricted feeding schedule, however, this turned out not to be an issue. Timed restricted feeding totally eliminated the difference in wheel-running activity between tPA^−/−^ and tPA^+/+^ mice. A strong bout of activity in the 3 h prior to food presentation was observed in both genotypes, with a compensatory decrease in activity during the latter half of the dark phase. There was a small but statistically significant difference in the change in body weight during the restricted feeding regime, with weight increasing slightly in tPA^+/+^ mice but not in tPA^−/−^ mice. It could be that increases in locomotor activity in tPA^−/−^ mice compared to baseline resulted in increased energy expenditure, however, given the short-term nature (~ 10 days) of the restricted feeding period we are reluctant to attribute much functional importance to this finding. It is also worth noting that the food anticipatory activity bout that appears during restricted feeding represents a consolidation of the increased daytime activity seen during fasting into a temporally coherent bout.

After a period of restricted feeding, we released mice into ad/lib feeding and constant darkness to assess the phase of the underlying nocturnal activity rhythm. We found no genotypic differences in free-running period, or phase at the time of the photoperiod transition, suggesting that SCN function was largely unaffected by restricted feeding. However, we did note that restricted feeding had an aftereffect on free-running period in the subsequent constant dark period, irrespective of genotype, in the form of a shortening of the period of the wheel-running rhythm. Since this rhythm is driven by the SCN, it suggests periods of restricted feeding may have a more subtle, long-lasting effect on the SCN.

As a result of seeing small differences in the change in body weight between genotypes during restricted feeding, we conducted a separate study on food intake. The protocol for this experiment differed from the locomotor activity studies in that the mice were housed in cages without running wheels, but which were designed for more accurate assessment of food intake. We found no genotypic differences in food intake during baseline conditions or during refeeding after a 48 h fast. However, total food intake was significantly reduced in restricted feeding as compared to ad/lib, and was reduced in tPA^−/−^ mice compared to tPA^+/+^. This manifested as a small decrease in fat mass in tPA^−/−^ mice that was not present in tPA^+/+^. These data do suggest that from an energetic standpoint the tPA^−/−^ mice have a slightly decreased ability to adapt to restricted feeding, but the lack of a difference in the fasted animals suggests that the difference is not metabolic but is more likely behavioral.

## Conclusions

Overall, the data presented here suggests that the effects of tPA on locomotor activity are primarily mediated by actions in the brain. The most likely route for these effects is through tPA’s regulation of mature BDNF production, and that tPA’s actions are stimulatory for wheel-running locomotor activity. Locomotor activity increases production of BDNF [26, 27]. From our studies, it is not possible to identify where in the brain tPA might be acting to influence these patterns of locomotor activity. The current literature regarding tPA’s functions in modulating hypothalamic-driven behaviors is sparse; further investigations into the location of tPA’s action in the brain may reveal important pathways that show divergence between locomotor activity driven by circadian rhythms and those driven by energetic demands.

## Additional file

Additional file 1: Activity profiles and body weight change in mice subjected to restricted feeding in a skeleton photoperiod. (PDF 603 kb)

## Abbreviations

BDNF: Brain-derived neurotrophic factor
FAA: food anticipatory activity
LD: light-dark
SCN: suprachiasmatic nucleus
tPA: tissue-type plasminogen activator
TrkB: Tropomyosin receptor kinase B
ZT: zeitgeber time
