# Supplementary material for: Regulation of Locomotor activity in fed, fasted, and food-restricted mice lacking tissue-type plasminogen activator
Source: BMC Physiol. 2018 Jan 25;18:2. doi: 10.1186/s12899-018-0036-0 (PMC5784530; doi:10.1186/s12899-018-0036-0)
Supplement: Additional file 1: — Activity profiles and body weight change in mice subjected to restricted feeding in a skeleton photoperiod. (PDF 603 kb) [file 12899_2018_36_MOESM1_ESM.pdf]

Supplementary File 1

Food anticipatory activity in tPA$^{-/-}$ mice with reduced light exposure (skeleton photoperiod).

Male tPA$^{+/+}$ and tPA$^{-/-}$ mice were entrained to a 12:12 LD cycle for at least 10 days. Light exposure was then limited to 15 minutes at the onset and offset of the active phase (ZT 12-12.25 and ZT23.75-0). Mice were then given two weeks to acclimate to the skeleton photoperiod, following which food was restricted for eight days. Note that because different running wheels were used for the skeleton photoperiod experiment than the 12:12 LD experiment, running wheel activity levels cannot be directly compared between the two experiments.

Food Restriction in a 12:12 skeleton photoperiod

In the skeleton photoperiod, the level of nocturnal activity was reduced in tPA$^{-/-}$ mice in skeleton photoperiod (LDsk) (Suppl Figs 1a, b) from ZT12-17 and 21 ($F_{(23,805)} = 4.57$, $p < 0.001$). As seen in LD, the pattern of locomotor activity was consistent between genotypes. It is important to note, however, that LD_{sk} activity cannot be compared directly to LD, as different types of wheels were used. On fast day one, tPA$^{-/-}$ mouse locomotor activity is less than tPA$^{+/+}$ (Suppl. Fig 1c) from ZT 13-18 ($F_{(23,785)} = 2.20$, p=0.001). There was no difference in activity levels on fast day two (Suppl. Fig 1d) at any time point ($F_{(23,805)} = 0.86$, p=0.653). Weight loss was consistent between genotypes following fast day two (tPA$^{-/-}$:-20.7% ± .97 and tPA$^{+/+}$: -18.8% ± 1.3)($t_{18} = -1.1466$, $p = 0.267$)(Suppl. Fig 2a). During restricted feeding days baseline differences in locomotor activity disappeared (Suppl. Fig 2c) ($F_{(23,805)} = 0.64$, p=0.899) as a result of increased tPA$^{-/-}$ activity. Following restricted feeding tPA$^{-/-}$ mice gained less weight than tPA$^{+/+}$ (tPA$^{-/-}$: .78± 1.12 tPA$^{+/+}$=4.7 ±1.43)($t_{17} = -2.1281$, $p = 0.048$) (Suppl. Fig 2b).

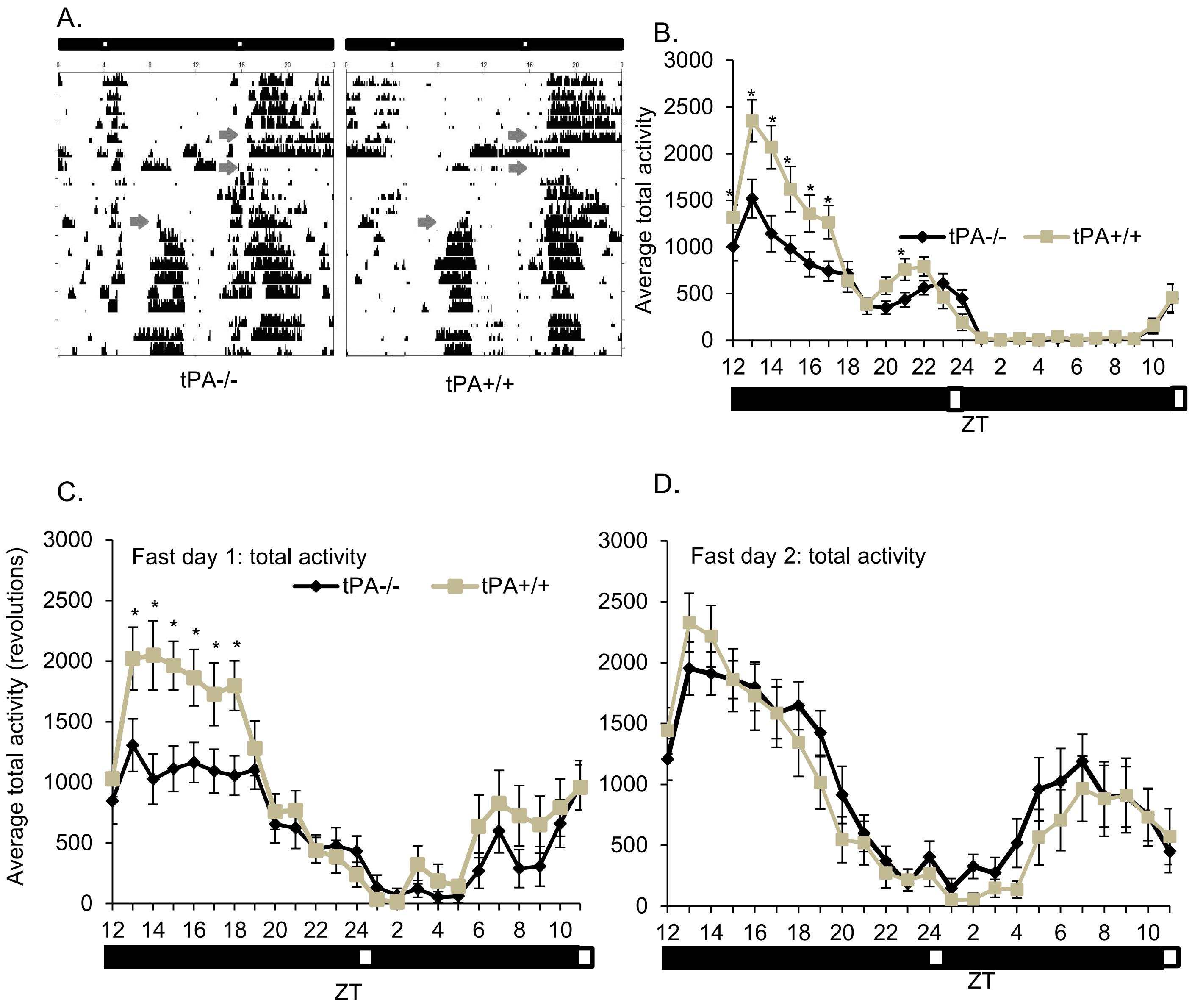

Supplementary Fig 1. Activity During LD_{sk} – Baseline and Fasting. Representative actograms (A) of food restriction protocol in $tPA^{-/-}$ and $tPA^{+/+}$ mice in LD_{sk}. Arrows indicate onset and offset of fast and beginning of restricted feeding. Fast includes food removal at ZT 12 and return 48 hours later at ZT 12. B. Average 24 hour activity profile (B), bar indicates dark phase; locomotor activity is higher in $tPA^{+/+}$ than $tPA^{-/-}$ at ZT 12-17 and 21 ($p<0.05$). During fast day 1 total locomotor activity (C) is higher in $tPA^{+/+}$ than $tPA^{-/-}$ at ZT 13-18 ($p<0.05$). During day 2 fast total locomotor activity (D) there are no differences between genotypes.

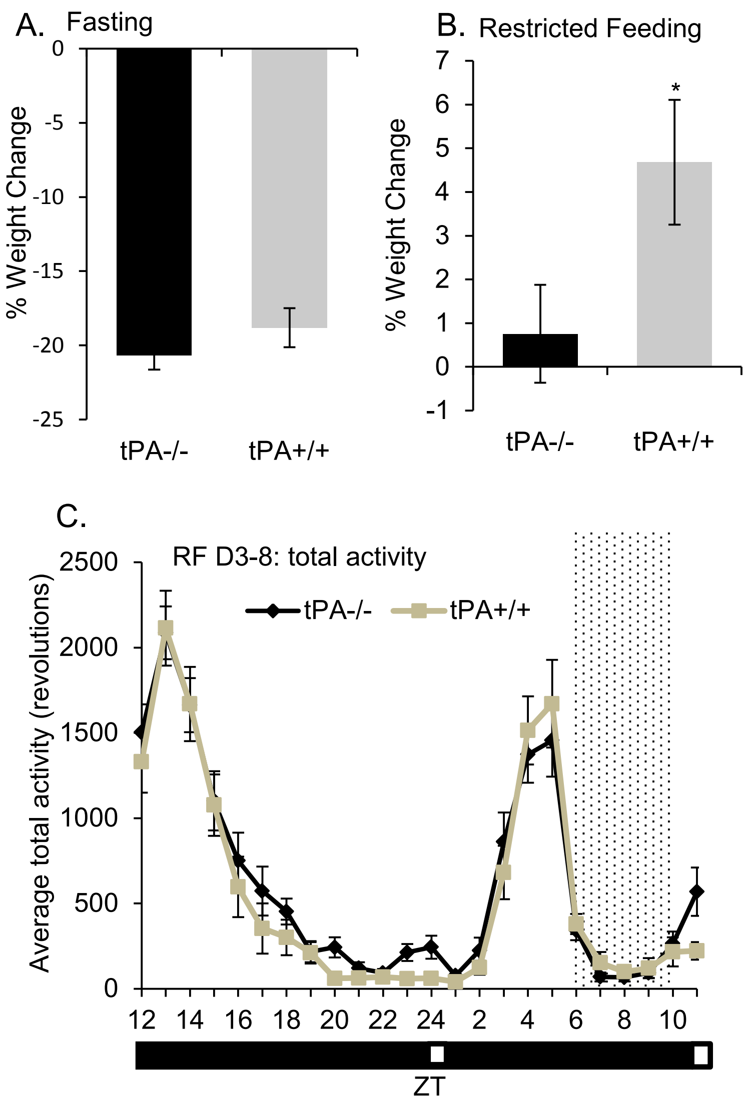

Supplemantary Fig 2. Body Weight Comparison. Weight changes between genotypes are consistent following a 48-hour fast (A); however, following restricted feeding (B) $tPA^{-/-}$ do not increase weight as much as $tPA^{+/+}$ ($p<0.05$). **Activity During LD_{sk} Restricted Feeding (C).** Activity profile averages from days 3 through 8 of restricted feeding. Bars indicate night phase and dots indicate food availability from ZT6-10. Total locomotor activity is not different between genotypes.
